# *Aspergillus*-SARS-CoV-2 Coinfection: What Is Known?

**DOI:** 10.3390/pathogens11111227

**Published:** 2022-10-25

**Authors:** Carlos Alberto Castro-Fuentes, María del Rocío Reyes-Montes, María Guadalupe Frías-De-León, Omar E. Valencia-Ledezma, Gustavo Acosta-Altamirano, Esperanza Duarte-Escalante

**Affiliations:** 1 Departamento de Microbiología y Parasitología, Facultad de Medicina, Universidad Nacional Autónoma de México, Avenida Universidad 3000, Ciudad Universitaria, Coyoacán, Mexico City 04510, Mexico; 2 Hospital Regional de Alta Especialidad de Ixtapaluca, Carretera Federal México-Puebla Km. 34.5, Pueblo de Zoquiapan, Ixtapaluca 56530, Mexico

**Keywords:** *Aspergillus*, SARS-CoV-2, CAPA, interaction, coinfection, susceptibility factors

## Abstract

COVID-19-associated pulmonary aspergillosis (CAPA) has had a high incidence. In addition, it has been associated with prolonged hospital stays, as well as several predisposing risk factors, such as fungal factors (nosocomial organism, the size of the conidia, and the ability of the *Aspergillus* spp. of colonizing the respiratory tract), environmental factors (remodeling in hospitals, use of air conditioning and negative pressure in intensive care units), comorbidities, and immunosuppressive therapies. In addition to these factors, SARS-CoV-2 per se is associated with significant dysfunction of the patient’s immune system, involving both innate and acquired immunity, with reduced CD4+ and CD8+ T cell counts and cytokine storm. Therefore, this review aims to identify the factors influencing the fungus so that coinfection with SARS-CoV-2 can occur. In addition, we analyze the predisposing factors in the fungus, host, and the immune response alteration due to the pathogenicity of SARS-CoV-2 that causes the development of CAPA.

## 1. Introduction

Severe acute respiratory syndrome (SARS)-CoV-2 was first identified in Wuhan, China, in December 2019, in groups of patients with pneumonia of unknown etiology. In January 2020, it was determined that the causal agent of this pathology was a new coronavirus allocated in the genus Betacoronavirus, which received the name SARS-CoV-2 because it was recognized as a sister clade to SARS-CoV [1]. On 11 February 2020, the World Health Organization (WHO) officially named the outbreak of coronavirus disease as Coronavirus Disease-2019 (COVID-19) [2]. Since the emergence of COVID-19, it has become a pandemic due to its high prevalence and rapid transmission [3]. It has been diagnosed in hundreds of millions of individuals and has killed millions worldwide [4].

The SARS-CoV-2 virus is composed of positive-sense single-stranded RNA, with a genome size of 27–32 kb. The virus has four main structural proteins, the spike protein (S), membrane (M), envelope (E), and nucleocapsid (N), which are necessary for the regulation of viral function and structure. The N protein helps the virus form the capsid and the entire viral structure correctly, while the S protein promotes the virus’s adherence to the host cell. The S protein consists of a large external domain, a single-channel transmembrane anchor, and a short intracellular tail. These three regions can precisely anchor the host cell. In these segments, the external domain contains two subunits, the receptor binding subunit S1 and the membrane fusion subunit S2. These subunits are located in the trimer-nail or crown structure, which is why it is named coronavirus [5,6,7]. SARS-CoV-2 glycoprotein S is cleaved by transmembrane serine protease 2 (TMPRSS2), producing two surface proteins, S1 and S2 [8].

It is known that in several coronaviruses, the S glycoprotein is cleaved at the boundary between the S1 and S2 subunits, which remain non-covalently bound in the prefusion conformation. The distal S1 subunit comprises the receptor binding domains, which contribute to the stabilization of the prefusion state of the S2 subunit, anchored to the membrane containing the fusion machinery [9].

The virus binds to the host cell via the S1 domain through the receptor-binding domain (RBD). It attaches to the angiotensin-converting enzyme 2 (ACE2) receptor to promote viral fusion and release the viral genome in host cells, which is necessary for producing new virions. Furthermore, it is known that the ACE2 receptor can be found on type II lung alveolar cells, small intestinal enterocytes, arterial and venous endothelial cells, and arterial smooth muscle cells in most organs [1].

Therefore, the virus has harmful effects, such as direct damage to pulmonary and extrapulmonary tissues, and the dysregulation of the immune response [10]. In addition, COVID-19 may be accompanied by infections caused by other microorganisms, including fungi, especially the *Aspergillus* genus [11,12].

In the last three years, there has been a significant increase in the incidence of aspergillosis among patients with COVID-19. Such an increase is due to lymphopenia and defective lymphocyte function, as well as hyperinflammatory reactions caused by systemic cytokines, which are associated with COVID-19 [13]. Likewise, the use of dexamethasone (DXM) for the treatment of COVID-19 patients causes immunosuppression [14], creating an additional risk factor for superinfections in these patients [15]. Likewise, it has been shown that patients with severe SARS-CoV-2 infection are affected by COVID-19-associated pulmonary aspergillosis (CAPA). However, Lai and Yu [16] mentioned that conventional risk factors for aspergillosis are not present in patients with CAPA.

Cases of CAPA have been reported since the start of the pandemic [17,18,19,20,21]. Table 1 shows the identified species of *Aspergillus* associated with CAPA in different parts of the world.

The reported incidence rate of CAPA among COVID-19 patients hospitalized in the intensive care unit (ICU) varies widely, from 2.4% to 35% [48,49]. However, the incidence of CAPA remains unclear [48,50,51]. In general, the incidence of CAPA has been higher in Europe than in North America. The variations in reported incidence may be due to several factors: host factors (ethnic and genomic aspects), environmental factors (facility ventilation systems, building materials, and nearby construction), and variations in clinical definitions and diagnostic approaches across institutions (including galactomannan (GM) testing and performance of bronchoscopy) [48,50,51].

On the other hand, in addition to immunosuppression, SARS-CoV-2 infection can also play an essential role in increasing susceptibility to invasive aspergillosis.

Thus, this review aims to identify which factors influence the fungus coinfection with SARS-CoV-2 and analyze the predisposing factors in the fungus, host, and the immune response alteration due to the pathogenicity of SARS-CoV-2 that cause the development of CAPA.

## 2. *Aspergillus* Features Influencing Susceptibility to COVID-19-Associated Pulmonary Aspergillosis (CAPA) Development

*Aspergillus* genus has unique characteristics that contribute to a higher incidence of CAPA. For instance, it is a ubiquitous saprophytic fungus to which humans are exposed daily in most parts of the world. *Aspergillus* is considered a nosocomial agent; however, there is no current uniform definition of what constitutes nosocomial aspergillosis. One of the main reasons for defining hospital-acquired aspergillosis is that the incubation period for *Aspergillus* is unknown [52]. In addition, the prolonged period of immunosuppression in high-risk patients, such as hematopoietic stem cell transplant (HSCT) recipients, as well as frequent hospital admissions and discharges, make it challenging to determine whether exposure to *Aspergillus* spores occurred during hospitalization or within the hospital setting. However, an invasive disease that occurs after one week of hospitalization is considered nosocomial. The most frequent nosocomial source of *Aspergillus* infection is contaminated air. Inhalation of airborne *Aspergillus* conidia is critical for infection [53]. Concentrations of *A. fumigatus* conidia in the environment are estimated to range from 1 to 100 conidia/m^3^. On average, an adult is likely to inhale more than 100 conidia per day [54]. Due to the relatively small size of the *A. fumigatus* conidia (2–3.5 µm), it has been widely assumed that these spores can reach the small airways and alveoli, a fundamental feature for their pathogenesis, making the alveoli the primary site of invasive pulmonary aspergillosis (IPA) [54,55,56,57,58]. Therefore, the continuous exposure of the fungus to local environmental conditions contributes to the invasion of the airways of critically ill patients infected with SARS-CoV-2. Likewise, host exposure to *A. fumigatus* conidia occurs frequently; however, the immune response in immunocompetent hosts is effective in removing them from the respiratory tract. In contrast, people with respiratory diseases, such as COVID-19 patients, are more likely to be colonized by *Aspergillus* during their hospital stay than those admitted for other reasons [59]. The ability of the *Aspergillus* spp. to colonize the airways is the first stage in the pathogenesis of invasive pulmonary aspergillosis. Colonization may be due to the special characteristics that patients have when they are admitted to the hospital; one of these differences is a prolonged stay in the intensive care unit (ICU), with a median of 14 days compared to 2 days in non-COVID-19 patients. The permanence of the patients in these areas is influenced by the fact that aspergillosis is acquired through the air, and the risk is related to the concentration of conidia in the air, a phenomenon determined by factors responsible for the release of conidia into the atmosphere as the presence of organic matter, deterioration of ventilation and air conditioning systems, heat and noise insulation material, and construction and demolition work in areas near or inside the hospital. Another factor related to the ability of *Aspergillus* spp. to colonize airways is the use of negative-pressure rooms [60]. During the current COVID-19 pandemic, it was recommended to place negative or normal pressure in intensive care wards to protect medical staff and patients. However, two recent studies reported a high incidence (26.3–33%) of pulmonary aspergillosis in patients infected with COVID-19 in rooms with negative pressure [17]. For this reason, Ichai et al. [61] showed that negative pressure in ICU wards could be the source of *Aspergillus* air contamination and thus increase the risk of opportunistic infections. When a switch was made to neutral or slightly positive pressure in the rooms, combined with environmental standards, cleaning protocols, and prophylactic antifungal treatments, *Aspergillus* was eradicated from room air. Contaminated respiratory equipment (humidifiers, nebulizers, oxygen canisters), which can be a source of infections, have also been investigated as potential sources of fungi in healthcare settings [62]. Likewise, in ICU patients with SARS-CoV-2 receiving dexamethasone (DXM) therapy, colonization by *A. fumigatus* (positive culture in tracheal aspirate) has been observed, and as a result these patients could be predisposed to develop CAPA, since steroids can create a favorable local environment for the in situ growth of *A. fumigatus* [63].

## 3. Host Factors Affecting Susceptibility to CAPA Development

Currently, the host risk factors associated with CAPA are not well understood. However, several studies include among the most critical factors the severity of COVID-19, age, previous respiratory diseases, chronic renal failure, chronic use of corticosteroids, neutropenia, and the prolonged treatment with corticosteroids or tocilizumab for COVID-19, in addition to thrombocytopenia, use of vasopressors before CAPA diagnosis, and the use of methylprednisolone at a daily dose of ≥40 mg, which is more likely to develop CAPA, as well as treatment with azithromycin [27,64,65,66,67]. Likewise, Dimopoulos et al. [67], in a review of papers published up to January 2021, describe the incidence of CAPA and associated comorbidities, suggesting that most CAPA cases occur essentially in severe COVID-19 patients.

In addition, pneumonia caused by viruses is known to increase patients’ susceptibility to bacterial and fungal coinfections, including invasive pulmonary aspergillosis (IPA), since respiratory viruses cause direct damage to the respiratory tract epithelium and make it difficult to eliminate the fungus through the ciliary system, which allows *Aspergillus* to invade the tissue. Furthermore, this leads to immune dysfunction or dysregulation, locally or systemically. Similarly, the degree of dysregulation associated with acute respiratory distress syndrome (ARDS) has shown that some patients develop marked immunosuppression, facilitating bacterial and fungal coinfection [13,68,69,70].

### 3.1. Comorbidities

In general, critically ill patients admitted to the ICU with comorbidities seem more susceptible to CAPA. This is very rare in non-severe cases, so the severity of COVID-19 is considered a risk factor. Among the most frequent comorbidities associated with CAPA are advanced age, diabetes, pulmonary diseases, cardiovascular diseases, hypertension, and solid organ malignancy [49,71,72]. However, other comorbidities associated with CAPA are also mentioned, including obesity, chronic respiratory diseases, and asthma [49].

Likewise, Gregoire et al. [64] cite other factors associated with CAPA, such as structural lung defects, severe lung damage during the course of COVID-19, and the use of broad-spectrum antibiotics. In addition, the authors found that patients with a history of cerebrovascular disease and hypertension also had a marked trend associated with CAPA [65]. Sagris et al. [73] have suggested a likely explanation for the increased severity of ischemic stroke observed in COVID-19 patients; the authors mention that a possible direct effect of SARS-CoV-2 on the brain may have a causal relationship with local brain ischemia and inflammation, in addition to brain tissue death, which causes, as a result, an excessive release of DAMP (damage associated molecular patterns), which in turn causes localized and global inflammation, and promotes the disruption of the blood–brain barrier. Thus, during the hyperinflammatory state of COVID-19, the overproduction of proinflammatory acute response proteins and adhesion molecules, together with activated circulating leukocytes, may result in the increase of the local inflammatory process in the ischemic brain. Although there are still not enough published statistical data, coinfections in SARS-CoV-2 patients have revealed that a significant number of hospitalized patients have developed secondary systemic mycoses, including aspergillosis, which cause serious complications, and even death. In severe cases, the risk of developing invasive fungal infections is high; this is not only due to the clinical situation of the patient and the need for invasive care, but also due to the immunological alterations caused by SARS-CoV-2 [74].

They also report that patients under treatment with immunosuppressants, as well as treatment with azithromycin (AZT) and hydroxychloroquine (HCQ), and DXM, showed a significant association with CAPA. They highlight that AZT has an immunomodulatory effect as it can inhibit neutrophils and the innate immune response, lowering the immune defense against *Aspergillus*. Further, its broad-spectrum antibiotic effect could alter the patients’ microbiota, thus promoting colonization by *Aspergillus*. In these patients, clusters of microbiomes have been observed to grow in the gut due to lymphopenia caused by infection [75].

Likewise, the use of corticosteroids to treat severe COVID-19 constitutes another risk factor associated with CAPA [71]. One study shows that long-term steroid treatment in doses greater than or equal to 16 mg/day of prednisone for at least 15 days was significantly more frequent in patients with CAPA than those without CAPA [23,76]. However, some studies have found no association between the use of these and the incidence of hospital-acquired fungal infections in patients with COVID-19. Feys et al. [71] and Salazar et al. [77] mention that the discrepancies can be attributed to the different cumulative doses of corticosteroids or the lack of statistical power to analyze the data.

The advanced age of COVID-19 patients shows significant implications for their immune systems. The most evident effect in elderly patients with cardiovascular diseases is that, due to the aging of their immune system, the imbalance within their innate and adaptive immune response generally worsens, causing a decrease in lymphocytes and an uncontrollable inflammatory reaction due to a cytokine storm. The result is an inadequate immune response, a lack of effective immune memory, and an inability to clear the virus. In addition, it causes injury to the lung epithelium and microvascular endothelium throughout the body, thus leading to ARDS and microvascular thrombosis, which could eventually favor the entry and development of *Aspergillus*, leading to high mortality among the elderly COVID-19 patients with cardiovascular diseases [78,79,80].

Additionally, various studies have shown a greater severity of COVID-19 in patients with diabetes mellitus. It is mentioned that patients infected with COVID-19 could predispose individuals to hyperglycemia, and by interacting with other risk factors, hyperglycemia could modulate the immune and inflammatory responses, predisposing patients to severe COVID-19 [81,82,83].

Furthermore, a study published by Sudhakar et al. [84] highlights obesity as a significant comorbidity in COVID-19 patients since it is a metabolic disorder with a deregulated immune and endocrine function, inferring that dysfunctional metabolism contributes to the fact that the mechanisms behind obesity are a risk factor with adverse outcomes in COVID-19 patients. The authors suggest that, in obese individuals, visceral adiposity expansion (VAT) leads to metabolic dysfunction, endoplasmic reticulum stress, immune cell infiltration, macrophage polarization to a pro-inflammatory phenotype, adipocyte cell death, and inflammation. It is also associated with the altered expression of adipokines and cytokines that cause systemic effects and dysfunction of endocrine and metabolic organs. They note that such a state of adipose tissue, particularly associated with multiple organs such as the lungs, vasculature, heart, and kidneys, may predispose obese subjects to adverse outcomes of COVID-19 infection.

In a large meta-analysis study by Wang et al. [85], evidence showed that COVID-19 patients with chronic obstructive pulmonary disease (COPD) have a risk of progression 5.9 times higher than patients without COPD. They also identified a higher risk of worsening in people with hypertension, diabetes, cardiovascular disease, or cerebrovascular disease, suggesting an opportunity for coinfections, including *Aspergillus*.

It is essential to mention that the comorbidities associated with the risk of acquiring CAPA are variable and depend on each study. Table 2 shows the main comorbidities related to CAPA, according to a review by Dimopoulos et al. [67], in different countries.

### 3.2. Changes in the Immune Response That Predispose to CAPA

Currently, there is no consensus to clearly define the risk factors associated with CAPA, and the results published to date should be taken with caution, since many studies are still under investigation. In addition, the variability in the diagnostic criteria used for the diagnosis of CAPA could lead to overdiagnosis, overtreatment, and overestimation of the true risk factors and associated outcomes, so significant predictors and prognostic factors may have been ruled out due to a lack of analyzed data [86].

**Figure 1:**
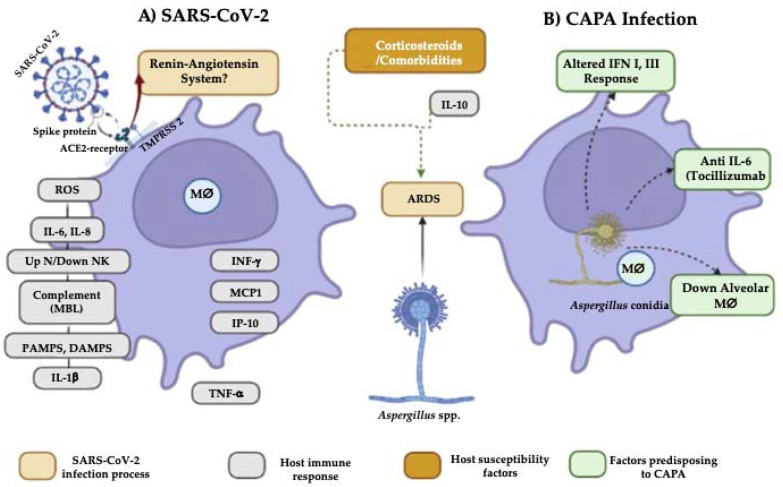
COVID-19-associated pulmonary aspergillosis (CAPA) infectious process. (**A**) Dysregulation of the renin-angiotensin system creates a pro-inflammatory environment in the host through reactive oxygen species (ROS), IL-6 and IL-8, pulmonary cell infiltrate by neutrophils, complement activation, and recognition of viral RNA through pathogen-associated molecular patterns (PAMPS), while damage-associated molecular patterns (DAMPS) stimulate large amounts of IL-1β and TNF-α. In addition, IFNγ, MCP1, and IP-10 are produced. This process causes acute respiratory distress syndrome (ARDS), which, together with corticosteroids, patient comorbidities, and *Aspergillus* in the environment, facilitates the fungal invasion of the host. (**B**) In addition, a deficient immune response from the host alters the IFN I and III production; likewise, medications and decreased alveolar macrophages favor susceptibility to CAPA.

It is known that influenza-associated pulmonary aspergillosis (IAPA) and CAPA share certain characteristics; however, IAPA has a higher incidence, most cases are observed in the first 48 h in the ICU, and it is more aggressive than CAPA. Moreover, the mechanism by which the intrinsic immune dysregulation caused by the influenza virus and SARS-CoV-2 occurs is unknown [71]. Recently, some hypotheses of the pathophysiology have been proposed that can explain the development of CAPA. The first hypothesis is related to the dysfunctional response of IFN (interferon) type I and III in severe COVID-19 patients (Figure 1), which has a fundamental role against *Aspergillus* spp., by promoting the response of type 1 T-helper cells against the fungus and driving type III IFN production, causing neutrophils to act against *A. fumigatus* [71]. The second hypothesis is related to IFN deregulation, together with cell exhaustion, particularly of alveolar macrophages, since they are the first cells to recognize inhaled conidia, being another possible cause of invasion by *Aspergillus* spp.

In IAPA, it is known that the effectiveness of phagocytosis against *Aspergillus* spp., associated with LC3 (Light Chain 3B) (which is a pathway for phagosome–lysosome fusion), is altered by the use of corticosteroids such as dexamethasone, hindering the activity of alveolar macrophages and consequently the production of anti-inflammatory cytokines such as IL-10 [87]; this alteration can also predispose the host to acquire CAPA [71]. Moreover, it is known that in severe cases of COVID-19, as well as in aspergillosis caused by *A. fumigatus* [88], in addition to CAPA, there are elevated levels of IL-6, which contributes to an increase in lung damage. Particularly in CAPA, tocilizumab (IL-6 receptor antagonist) has been used to reduce its expression; however, its administration has not shown efficacy in resolving the disease since it does not regulate the basal production of this cytokine and prevents the Th17 protective response and effector functions of phagocytes [13,16,35,77].

Other risk factors that predispose to CAPA are deregulation of the renin-angiotensin system and/or the kallikrein-kinin system produced by the binding of SARS-CoV-2 to the ACE2 receptor; however, its mechanism is unknown [13]. As part of the innate immune response, pathogen-associated molecular patterns (PAMPS), damage-associated molecular patterns (DAMPS), and pattern recognition receptors (PRR) are present in macrophages, dendritic cells, and neutrophils, and constitute the first line of defense against the virus. In this sense, a risk factor is due to cellular deterioration caused in NK cells, whose function is the direct elimination of fungal pathogens and stimulation of other cells such as neutrophils [89], which has been related to increased susceptibility to co-infections [77]. Platelets represent another predisposing factor since they are considered key regulators of the innate immune response, and it has been shown that an increase in the platelet count is associated with the severity of the disease in patients with COVID-19. This is due to the fact that platelets express ACE2; furthermore, in vitro exposure to SARS-CoV-2 has been observed to potentiate platelet activation and aggregation, resulting in vascular complications seen in patients with COVID-19 and increased susceptibility to co-infections [77].

Moreover, the production of IL-1β in high concentrations leads to a pro-inflammatory environment, making fungal pathogenesis permissible. This is due to the production of DAMPS, which are proposed as a possible immunomodulatory strategy in CAPA because they promote and exacerbate the immune and inflammatory response leading to lung injury, particularly due to an increase in the inflammatory reaction in experimental aspergillosis [13], predisposing COVID-19 patients to secondary *Aspergillus* infection.

Another host susceptibility factor is lymphopenia, which, together with the pro-inflammatory environment and the cytosine storm, can have an impact on the functionality and efficacy of the immune response [90], which leads to, as a result, the development of CAPA in individuals who present some type of comorbidity. Although efforts have allowed us to learn a little more about this coinfection, the pathophysiological processes that originate it have yet to be discovered.

### 3.3. Influence of Antifungals on the Aspergillus-SARS-CoV-2 Interaction

In invasive aspergillosis (AI), it is known that early intervention with an appropriate antifungal agent can contribute to patient improvement, so the use of voriconazole is recommended as the first line of defense [13]. Voriconazole has been widely used in CAPA; however, the interaction between voriconazole and drugs used in the treatment of COVID-19 (hydroxychloroquine, azithromycin, and protease inhibitors, such as lopinavir/ritonavir) has been reported to cause cardiac events [91,92]. As a result, the treatment is not effective for patients [93], demonstrating its low effectiveness, which has also been reported for itraconazole [13].

Furthermore, *Aspergillus fumigatus* and *A. flavus* are the opportunistic pathogens most commonly reported as etiological agents of CAPA [16]. Likewise, it is known that *A. flavus* has less sensitivity to voriconazole, amphotericin B, and 5-fluorocytosine [94], so the use of a single antifungal is not the best approach to show good results; particularly in the case of CAPA, the combination with other antifungals is promising [95]. Moreover, antifungals such as isavuconazole show a more favorable pharmacokinetic profile, and the toxicity is lower compared to voriconazole [13]. However, more studies are required to evaluate the usefulness of isavuconazole in CAPA.

Echinocandins have also been used for the treatment of CAPA, particularly caspofungin, which produced better results in these patients [96]. In addition, it is proposed that its use can prevent fungal infections and reduce severe cases of COVID-19, mainly in patients with hematological malignancies and who are in the ICU [97].

Due to the resistance to antifungals of some species of *Aspergillus* spp., clinical trials of new antifungals are currently being studied as therapeutic tools, which can be very useful in treating CAPA [98]. These antifungals include opelconazole, fosmanogepix, ibrexafungerp, olorofim, and rezafungin [71]. However, most are in phase III, so they are not yet available on the market. In addition, the efficacy of prophylactic treatment in the first days of stay of COVID-19 patients in the ICU has been evaluated, highlighting the efficiency of prophylaxis with posaconazole, which allows the reduction of CAPA cases by >90% [99]. No significant impact on survival was demonstrated; however, it could be considered an option since the drug–drug interaction, as occurs with voriconazole, is less due to the fact that it is metabolized by a different pathway.

## 4. Conclusions

It has been shown that the incidence of CAPA is influenced by several elements, including host factors, *Aspergillus* intrinsic features, aspects caused by SARS-CoV-2, and environmental components. Among the main host risk factors for acquiring CAPA are the severity of COVID-19, age, previous respiratory diseases, chronic renal failure, chronic use of corticosteroids, neutropenia, and prolonged treatment with corticosteroids or tocilizumab for COVID-19. In addition, thrombocytopenia, using vasopressors before CAPA diagnosis, using methylprednisolone, and treatment with azithromycin are also relevant aspects, as are relevant comorbidities.

Additionally, the main aspects of the fungus associated with CAPA are its ubiquity, the fact that it is considered a nosocomial organism, the size of its conidia, and the ability of *Aspergillus* spp. to colonize the respiratory tract.

Likewise, the environmental factors associated with CAPA are renovations of hospital facilities, the use of air conditioning, and negative pressure in intensive care units. Finally, the respiratory viral infection caused by SARS-CoV-2 produces multiple lung changes that create deregulation of the host’s immune system, facilitating the invasion of *Aspergillus*, which results in CAPA.

Despite a large number of CAPA cases in the literature, there is little information about the factors involved in the *Aspergillus*-SARS-CoV-2 interaction. Therefore, it is necessary to expand studies to elucidate the host’s role and the microorganisms participating in CAPA.

## Figures and Tables

**Table 1:** *Aspergillus* species associated with COVID-19-associated pulmonary aspergillosis.

Species	Country (Cases Number)	Identification Method	Reference
*A. fumigatus/A.* section *Fumigati*	Italy (9)	Culture from BAL and AT	[14]
*A. fumigatus/A.* section *Fumigati*	Germany (4)	Culture from BAL, AT, and PCR	[18]
*A. fumigatus/A.* section *Fumigati*	Netherlands (6)	Culture from BAL, AT, and sputum	[21]
*A. fumigatus/A.* section *Fumigati*	Italy (1)	Culture from BAL	[22]
*A. fumigatus/A.* section *Fumigati, A. niger/A.* section *Nigri, A. flavus/A.* section *Flavi*	Italy (NA)	Culture from BAL	[23]
*A. fumigatus/A.* section *Fumigati*	France (1)	Culture from AT (X2)	[24]
*A. fumigatus*	United Kingdom (NA)	Respiratory secretions (BAL fluids, NBL fluids, TA, and secretions) were subjected to GM testing, *Aspergillus*-specific PCR, microscopic examination, and mycological culture. A limited selection of serum or BAL fluid samples was also subjected to *Aspergillus*-specific LFD testing.	[25]
*A. fumigatus/A.* section *Fumigati*	France (NA)	Culture from BAL and AT	[26]
*Aspergillus* spp.	France (NA)	Culture from BAL and AT	[27]
*A. fumigatus/A.* section *Fumigati, A. flavus/A.* section *Flavi, A. calidoustus/A.* section *Usti*	France (NA)	Culture from BAL, AB, and AT	[28]
*A. fumigatus (TR34/L98H mutation)/A.* section *Fumigati*	France (1)	Culture from AT	[29]
*A. fumigatus/A.* section *Fumigati*	France (NA)	Culture from BAL and AT	[30]
*A. fumigatus/A.* section *Fumigati*	Denmark (NA)	Culture from BAL and AT	[31]
*A. fumigatus/A.* section *Fumigati*	Germany (2)	Culture from BAL	[32]
*A. fumigatus/A.* section *Fumigati*	Swiss (NA)	Culture from AT	[33]
*A. flavus/A.* section *Flavi*	France (1)	Culture from AT	[34]
*A. fumigatus/A.* section *Fumigati, A. citrinoterreus/A*. section *Terrei, A.* *lentulus/A.* section *Fumigati*	Spain (NA)	Culture from BAL	[35]
*A. fumigatus (TR32/L98H mutation)/A.* section *Fumigati*	Netherlands (1)	Culture from BAL and AT	[36]
*A. fumigatus (TR34/L98H mutation)/A.* section *Fumigati*	Ireland (1)	Culture from AT	[37]
*A. flavus/A.* section *Flavi, A. fumigatus/A.* section *Fumigati, A. terreus/A.* section *Terrei*	Pakistan (NA)	Culture from BAL, AT, and sputum	[38]
*A. fumigatus/A.* section *Fumigati*	Austria (1)	Culture from AT	[39]
*A. fumigatus/A.* section *Fumigati, Aspergillus* spp., *A.* *flavus/A.* section *Flavi, A. niger/A.* section *Nigri*	Mexico (NA)	Culture from AT	[40]
*A. fumigatus/A.* section *Fumigati, A. flavus/A.* section *Flavi*	Belgium (7)	Culture from BAL and AT	[41]
Unidentified	Brazil (1)	Sequencing identifying *A. penicillioides*	[42]
*A. fumigatus, A. niger*, *A. flavus*	Spain (NA)	ITS1-5.8S-ITS2 amplification by PCR and Sanger sequencing	[43]
*A. fumigatus/A.* section *Fumigati*	Australia (1)	Culture from AT (X3)	[44]
*A. fumigatus/A.* section *Fumigati, A. flavus/A.* section *Flavi, A. terreus/A.* section *Terrei*	Netherlands (NA)	Culture from BAL	[45]
*Aspergillus* spp.	Mexico (NA)	Culture from BAL	[46]
*A. fumigatus/A.* section *Fumigati*	France (NA)	Culture from BAL and AT	[47]

BAL: bronchoalveolar lavage; AT: tracheal aspirate; NBL: non-bronchoscopic lavage specimens; GM: galactomannan; PCR: polymerase chain reaction; LFD: lateral flow device; BDG: β-D-glucan; AB: bronchial aspirate; NA: not available.

**Table 2:** Risk factors for COVID-19-associated pulmonary aspergillosis in different countries.

Country	Number of Cases	Comorbidities
Italy	108	Obesity, AH, DM, coronary disease, COPD, CRF, hemodialysis, cerebrovascular disease, malignancies, solid organ transplant, chronic steroid treatment, and atrial fibrillation.
France	615	AH, obesity, DM, BA, cardiac disease, gout, thyroid cancer, MDS, Hashimoto disease, hyperlipidemia, cancer, hemopathy, CRF, kidney transplant recipient, HIV, steroid treatment, COPD, dialysis, stroke, CHF (NYHA classification 3–4), arrhythmias, CRF.
Belgium	20	AH, DM, hypercholesterolemia, CRF, obesity, AML, HIV.
China	152	DM, AH, heart disease, COPD, CRF.
Germany	21	AH, COPD, DM, obesity, OSA, pulmonary fibrosis.
Netherlands	74	Cardiomyopathy, COPD, BA, DM, AH, chronic steroid treatment, neutropenia, stem cell transplant, immunodeficiency, DM, CRF.
Austria	1	COPD, OSA, Obesity, DM, AH, and cardiac disease.
Netherland	74	Reflux, polyarthrosis.
Ireland	1	DM, AH, hyperlipidemia, obesity.
Brazil	1	AH, DM, CRF.
Pakistan	23	DM, AH, atrial myxoma, recent stroke.
Switzerland	118	AH, DM, obesity, pulmonary fibrosis, BA.
Spain	239	MDS, HIV, DM, COPD, ankylosing spondylitis, acquired hemophilia A, hypothyroidism, CLL, cardiac disease, AH, BA, obesity, CRF, non-alcoholic fatty liver disease, and CNS disease.
Denmark	8	AH, BA.
UK	916	DM, AH, CRF, obesity, cancer, CRF malignancy, hyperlipidemia, cardiac and vascular disease, and autoimmune disorders.
USA	46	Atrial fibrillation, COPD, AH, OSA, DM, CRF, coronary disease, CHD, ESRD, nephrectomy, vasculitis, junctional tachycardia, bipolar disorder, hypercholesterolemia, obesity, hypothyroidism, gastric ulcer, atherosclerosis, and sarcopenia.

AH: arterial hypertension; DM: diabetes mellitus; COPD: chronic obstructive pulmonary disease; CRF: chronic renal failure; BA: bronchial asthma; AML: acute myeloid leukemia; HIV: human immunodeficiency syndrome; OSA: obstructive sleep apnea; MDS: myelodysplastic syndrome; CLL: chronic lymphocytic leukemia; CRF: chronic respiratory failure; CNS: central nervous system; CHF: congestive heart failure; NYHA: New York Heart Association; CHD: congenital heart disease; ESRD: end-stage renal disease.

## Data Availability

Not applicable.
